# Fracture of the Clavicle

**Published:** 1874-09

**Authors:** A. R. Kilpatrick

**Affiliations:** Texas


### FRACTURE OF THE CLAVICLE.

BY A. R. KILPATRICK, M.D., OF TEXAS.

This is a fracture met with oftener than any other, and having seen in sundry medical journals different plans of bandaging and treating it, I have concluded to add one not enumerated. The Philadelphia *Medical Times*, for March 7th, 1874, has a plan laid down by Dr. Lewis A. Sayre, to use broad strips of adhesive plaster, what he calls "my adhesive plaster dressing for fractured clavicle."

We all know that bandages fail to hold the shoulder and fractured bone in position, unless the patient is closely confined. Adhesive plaster becomes detached and loose in a day or two, requiring renewal. The indications are to keep the shoulder elevated, and thrown rather posteriorly, or else the ends of the fractured clavicle will miss opposition, and the bones become lapped. I have seen cases of such bad surgery requiring re-fracture, and treatment *de novo.*

I always use the *yoke-splint,* made of wood of a light quality, yet strong and firm. The splint should extend behind the neck, from an inch beyond one shoulder point to the same distance beyond the other. These splints are, in all, outfits of wooden splints, and if one of an unusually large size or small size is needed, any good workman can easily shape one out of poplar or maple wood. Have it padded well inside with cotton, and a notch cut to fit the neck. Have strips of garter stuff, or soft leather, tacked on each end to pass under the arms, and a buckle on one end so as to fasten and hold the shoulders steady.

Having the yoke-splint ready, reduce the fractured ends and adjust the splint over the shirt or under-dress of the patient, and turn him or her loose. I have treated many cases with the yoke-splint, and allow the patient, be it child or adult, to walk about and take moderate exercise, and I have met with no failures, nor any bad cures. As the case progresses, the splint should be noticed, as it sometimes is loosened by the patient, or the strips under the armpits may become loosened and require tightening. If the strips cause soreness or abrasion under the axillæ, carded cotton, or bits of old soft quilt, can be so placed as to give relief.

The splints are scooped or troughed out similar to a bread-tray, so as to fit the curvature of the shoulders, and the padding inside is fastened with tacks.

---

*Remedy for Chronic Hoarseness.*—An eminent physician of Philadelphia contributes the following: In chronic hoarseness, arising from thickening of the vocal chords and adjacent membrane, the ammoniated tincture of guaiacum is often a very efficacious remedy. It may be appropriately mixed with equal parts of the syrup of senega, and a teaspoonful of the mixture given two or three times a day.—*American Practitioner.*

